# Placental stem cells-derived exosomes stimulate cutaneous wound regeneration *via* engrailed-1 inhibition

**DOI:** 10.3389/fbioe.2022.1044773

**Published:** 2022-12-09

**Authors:** Yan Zhang, Liyan Shi, Xiuying Li, Yang Liu, Guokun Zhang, Yimin Wang

**Affiliations:** ^1^ China-Japan Union Hospital of Jilin University, Changchun, China; ^2^ Jilin Province People’s Hospital, Changchun, China; ^3^ Institute of Antler Science and Product Technology, Changchun Sci-Tech University, Changchun, China

**Keywords:** placental MSCs, exosomes, wound regeneration, SCAR, engrailed-1

## Abstract

**Introduction:** Skin wounds generally heal by scarring, a fibrotic process mediated by the Engrailed-1 (EN1) fibroblast lineage. Scar is detrimental to tissue structure and function, but perfect healing in clinical settings remains to be explored. Recent studies have shown that mesenchymal stem cell (MSC) transplantation can reduce scarring

**Methods:** Here, we investigated the effects of placental MSCs (pMSCs) and exosomes derived from pMSCs (pMSC-exos) on wound healing using a full-thickness rat model.

**Results:** The results showed that placental MSCs significantly accelerated the wound healing rate. Moreover, placental MSCs improved the quality of wound healing, including regenerating cutaneous appendages (hair follicles and sebaceous glands), decreasing collagen I and increasing collagen III, and improving collagen pattern (basket-wave-like) in the healed skin. placental MSCs treatment also increased the regeneration of blood vessels. Importantly, all these listed effects of placental MSCs were comparable to those of exosomes derived from pMSCs, but significantly stronger than those of adipose MSC-derived exosomes (aMSC-exos). Further studies showed that the effects of placental MSCs and exosomes derived from pMSCs on wound regeneration may be mainly achieved *via* the down-regulation of the Yes-associated protein signaling pathway, thus inhibiting the activation of EN1.

**Discussion:** In summary, placental MSCs could effectively stimulate wound regeneration, and their effect could be achieved through their exosomes. This suggests that exosomes derived from pMSCs treatment could be used as a novel cell-free approach to induce wound regeneration in clinical settings.

## 1 Introduction

The fibrotic scar on the skin generally results from adult wound healing (Martin, 1997; Walmsley et al., 2015). Scar differs from normal skin in that it lacks cutaneous appendages (hair follicles, sebaceous glands and sweat glands) and possesses an extracellular matrix (ECM) with dense, parallel fibers (Gurtner et al., 2008; Eming et al., 2014; Sun et al., 2014). The scar can cause psychological and physiological distress, and the costs of managing it and its sequelae are overwhelming (Sen et al., 2009; Mascharak et al., 2021). A scar-free wound healing would be desirable (Mascharak et al., 2021), but it has not been achieved thus far despite decades of research. Recent studies indicated that the scarring is mainly mediated by the Engrailed-1 (EN1) fibroblast lineage, and preventing EN1 activation can promote wound regeneration with the recovery of cutaneous appendages and ultrastructure (Rinkevich et al., 2015; Mascharak et al., 2021). Recently, it was reported that up-regulation of the Yes-associated protein (YAP) signaling pathway induced EN1 activation in wound healing and that promoting YAP degradation blocks the progression of scarring in wound healing (Mascharak et al., 2021). Thus, intervention of the YAP signaling pathway may be an effective way to inhibit EN1 activation, are proposed therapeutic approaches to reduce scarring and even achieve scarless wound healing.

Among all available therapeutic approaches to promote wound regeneration, mesenchymal stem cell (MSC) transplantation has been recognized as a promising strategy in recent decades (Ojeh et al., 2015; Fang et al., 2016; Duan et al., 2020). The advantages of MSC application include relatively easy expansion *in vitro*, and the ability to home in the injury site and differentiate into specific cell types required for tissue regeneration (Abdelwahid et al., 2016; Han et al., 2019; Duan et al., 2020; Rong et al., 2020). However, the successful application of general adult MSCs is limited because they retain epigenetic changes even after reprogramming (Wechsler et al., 2021). In contrast, placental MSCs (pMSCs) own stronger differentiation potential (Yi et al., 2020; Maraldi and Russo, 2022), which have been demonstrated own immune-modulating properties (Abumaree et al., 2017) and capable to induce regeneration of spinal cord (Jiang et al., 2017), heart (Huynh, 2019), skeleton (Longo et al., 2012), *etc.* However, up to now, few studies have investigated the effect of pMSCs on wound healing. The effect of pMSC-derived exosomes (pMSC-exos) on wound healing was even less conducted, even though they have powerful advantages in stem cell therapy. Exosomes are small vesicles secreted by cells, with a diameter of about 30–150 nm, and carry a variety of paracrine “cargoes,” such as DNA, miRNA, functional proteins, and lipid signals. These “cargoes” can mediate communication between cells in a paracrine manner. Recipient cells normally take up exosomes through phagocytosis, endocytosis, or fusion, thereby receiving the cargo. Therefore, exosomes are considered to be the main factor of paracrine components in the efficacy of stem cell therapy (Batrakova and Kim, 2015; Pegtel and Gould, 2019).

The present study investigated the effects and mechanism of pMSCs and pMSC-exos on wound regeneration using a full-thickness rat model. We found that pMSC-exos had comparable effects to pMSCs, which improved the healing rate and quality of the rat wound models. Furthermore, we found that the effects of pMSCs were likely achieved through EN1 inhibition by down-regulating YAP signaling pathway. These results above suggest that pMSC-exos present a novel strategy for inhibiting scarring and improving wound regeneration during wound healing in clinical settings.

## 2 Materials and methods

### 2.1 Cell culture and characterization

Placental tissue from pregnant rats was cut into small pieces and rinsed extensively with 5% penicillin/streptomycin in PBS to remove blood. Placental pieces were then placed in petri dishes and cultured in a DMEM containing 10% fetal bovine serum (FBS; Gibco, United States), and 1% penicillin/streptomycin (BI, Israel) under 37°C, 5% CO_2_, and saturated humidity. The medium was replaced every 3 days pMSCs were trypsinized with 0.25% and passaged at a ratio of 1:3 when they reached 80% confluence. pMSCs (passages 3–5) were stored at −80°C and used for subsequent experiments.

pMSCs were characterized through profiling of markers (CD34, CD45, CD73, CD90, and CD105) *via* IF staining. Primary pMSCs were fixed with methanol and incubated with primary antibodies (Supplementary Table S1). Then the cells were stained with Cy3-labeled secondary antibodies. DAPI (Beyotime, China) staining was carried out for nuclear visualization, and finally, the cells were photographed under a fluorescence microscope (Olympus, Japan).

Rat adipose MSCs (aMSCs) were cryopreserved in our laboratory and were cultured in a DMEM (BI, Israel) containing 10% FBS and 1% penicillin/streptomycin under 37°C, 5% CO_2_, saturated humidity.

### 2.2 Exosome preparation

The pMSC-exos were prepared according to our previous study protocol (Duan et al., 2020; Zhang et al., 2021a; Zhang et al., 2021b). Briefly, the cells were cultured in the FBS-containing DMEM, and the medium was replaced with a serum-free medium (Hyclone, United States) when they reached around 80% confluence. After another 48 h, the culture supernatant was collected and centrifuged as follows: 2,000 g for 10 min (removing dead cells) → 10,000 g for 30 min (removing debris) → 100,000 g for 70 min (collecting exosomes). The prepared pMSC-exos were stored at −80°C until use (re-suspend using PBS). The protein concentration of the exosomes was quantified using a bicinchoninic acid protein assay kit (Solarbio, Beijing, China). Characterization of pMSC-exos was performed using transmission electron microscopy, NanoSight NS300 (Malvern Instruments, United Kingdom), and exosome marker (TSG101, CD9, and CD63) (Shi et al., 2021).

The preparation process of aMSC-derived exosomes (aMSC-exos) was the same as that of pMSC-exos.

### 2.3 Cutaneous-wounded model creation and treatment

Eight-week-old rats (Liaoning Changsheng, China) were used for the animal experiment, which was approved by the Animal Ethics Committee of Changchun Science-Technology University (No. CKARI2020013). The rats were anesthesia using 3% pentobarbital sodium (30 mg/kg), and full-thickness skin was then excised using a 12 mm skin biopsy punch (Acuderm, United States) (Duan et al., 2020; Zhang et al., 2021a; Zhang et al., 2021b; Zhang et al., 2021). Wounded rats were divided into four groups (16 rats/group): aMSC-exos (50 μg), pMSCs (2 × 10^6^ cells), pMSC-exos (50 μg), and control (PBS). Cells or exosomes were topically administered weekly *via* injection around the wound margins (25 μl × 4 injections). The wounds were photographed weekly, and the wound area was calculated using ImageJ software. The healed skin was harvested on 28 days post wounding (DPW) for histological and molecular analyses.

### 2.4 Histology

Healed skin was fixed with 10% formaldehyde for 72 h and embedded in paraffin. Following the manufacturer’s instructions, 5.0 µm sections were prepared and stained with HE, Masson, and Sirius Red; and then photographed under the Microscope (Precipoint M8, Germany; BX53, Olympus, Japan). The number of cutaneous appendages, including hair follicles and sebaceous glands, were counted per 20× high-power field (HPF) in the healed skin according to the image of HE staining. The percentage of collagen fibers (blue area) in the healed skin was quantitated per 20× HPF using Image-Pro Plus software according to the image of Masson staining. The ratio of collagen I (red area) to collagen III (green area) in the healed skin was quantitated per 20× HPF according to the image of Sirius Red staining.

IF staining was performed on paraffin sections with specific primary antibodies (CD31 and EN1; Supplementary Table S1). Then the sections were stained with Cy3-labeled secondary antibodies. DAPI (Beyotime, China) staining was carried out for nuclear visualization, and finally the sections were photographed under a Microscope (Olympus, Japan). The positive expression number of cells was quantified using Image-Pro Plus software.

### 2.5 Quantitative real-time polymerase chain reaction (qRT-PCR)

Total RNA of healed skin was isolated using Trizol (Invitrogen, United States) and used for cDNA synthesis *via* a cDNA Synthesis Kit (Takara, Japan). The qRT-PCR was performed to determine the mRNA levels of EN1 (Forward: 5′-CTG​GGG​AAG​TCA​AAC​CCC​TC-3′, Reverse: 5′-GCA​GGG​GCT​GTT​CCT​TTT​TG-3′) using SYBR Green Master (Roche, Switzerland) in ABI 9700 Detection System. Results of mRNA quantification were normalized against GAPDH and calculated using the ^ΔΔ^Ct method. All reactions were performed in triplicate.

### 2.6 Western blot

The healed tissues were lysed using RIPA reagent (Invitrogen, United States) supplemented with proteinase inhibitors. The proteins were loaded and separated *via* polyacrylamide SDS gel. Following electrophoresis, the protein bands were transferred onto polyvinylidene fluoride membranes (Millipore, MA), blocked in 5% (w/v) non-fat milk, and incubated with the primary antibodies overnight and the secondary antibody (horseradish peroxidase-conjugated IgG [H + L]) for 2 h. The primary antibodies are listed in Supplementary Table S1. Peroxidase activity was detected by enhanced chemiluminescence and captured with Tanon-5800 (Tanon, China). The target protein bands were quantified using ImageJ software and normalized to the signal intensity of GAPDH.

### 2.7 Statistical analysis

Data are reported as means ± SEM (*n* ≥ 3). Statistical analysis was performed using GraphPad Prism. Comparisons between groups were performed using Student t-tests; *p* < .05 (*), <.01 (**), <.001 (***), or <.0001 (****) was considered statistically significant. Skin appearance and histological evaluations were performed in a blinded fashion.

## 3 Results

### 3.1 Characterization of pMSCs and preparation of pMSC-exos

**FIGURE 1:**
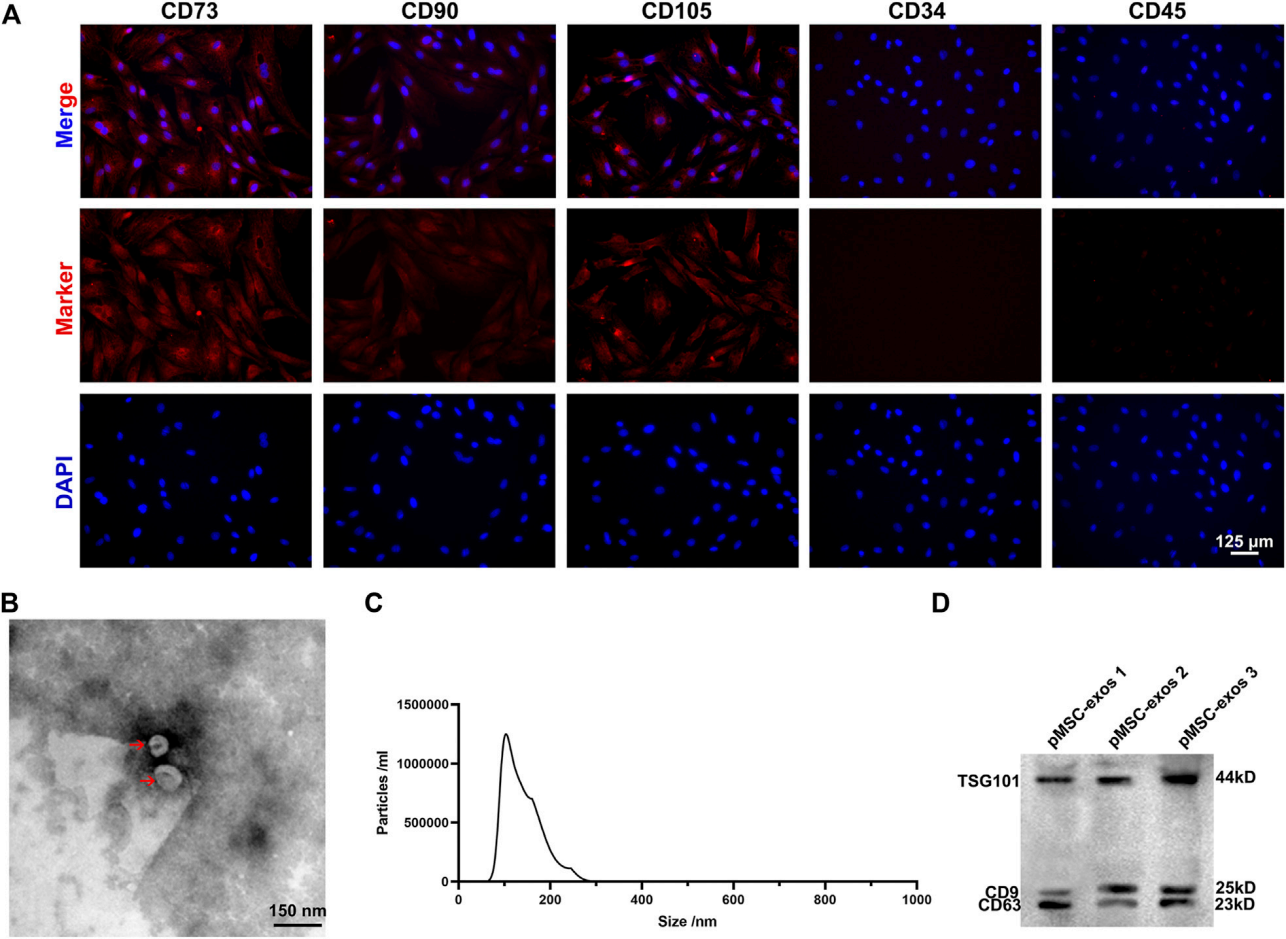
Characterization of pMSCs and pMSC-exos. **(A)** The cells isolated from the placenta were expressed positively for CD73, CD90, and CD105, and negatively for CD34 and CD45 via IF staining. **(B)** Morphology of pMSC-exos via transmission electron microscopy. **(C)** Particle size of pMSC-exos via NanoSight. **(D)** Detection of exosome markers (TSG101, CD9 and CD63) in pMSC-exos via western blot assay. MSC, mesenchymal stem cell; pMSCs, placental MSCs; pMSC-exos, pMSC-derived exosomes; IF, immunofluorescence.

pMSCs were identified *via* IF staining negative for CD34 and CD45, and positive for CD73, CD90 and CD105 (Figure 1A). The pMSC-exos were prepared using ultra-high-speed centrifugation and characterized using transmission electron microscopy, NanoSight and western blot analysis. Results showed that pMSC-exos exhibited a round-shaped morphology (Figure 1B) with an average size of 110 nm diameter (Figure 1C); and expressed exosome markers, including TSG101, CD9, and CD63 (Figure 1D). In summary, the isolated pMSC-exos has high purity and can be used in the following experiments.

### 3.2 pMSC-exos accelerate wound healing rate in rat

**FIGURE 2:**
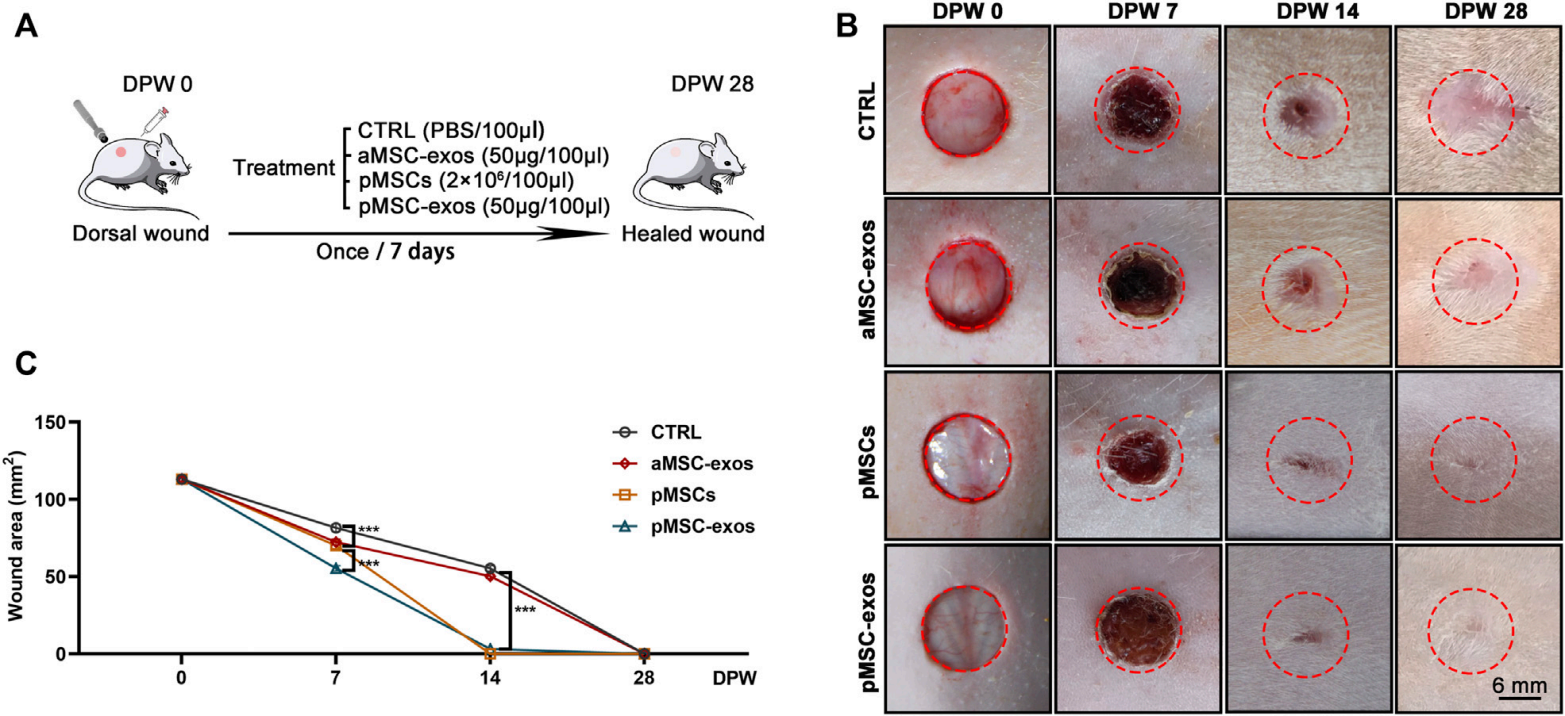
pMSC-exos accelerate wound healing rate. **(A)** Schematic drawing of animal experimental design. **(B)** Morphological changes of the wounds during the healing period. **(C)** Quantitative evaluation of changes in wound area during the healing period. Mean ± SEM; ***p < .001, *n* = 8. CTRL, control; aMSC-exos, adipose MSC-derived exosomes; DPW, days post wounding.

The full-thickness wounds in rats were created to evaluate the effects of pMSC-exos on wound healing (Figure 2A). Results showed that on 7 DPW, the wound areas (mm^2^) showed a significant difference in size in different groups: CTRL (81.71 ± 3.34) > aMSC-exos (72.38 ± 2.96) > pMSC-exos (70.21 ± 2.87) > pMSCs (55.42 ± 2.26). On DPW 14, wounds in the pMSCs and pMSC-exos groups were essentially closed, but those in the CTRL (55.42 ± 2.62) and aMSC-exos groups (50.27 ± 2.05) were on the way, and finally closed on POD 28 (Figures 2B, C). Laterally, the outcome of the wounds of the CTRL group was distinct, hairless scars; the wounds of aMSC-exos, pMSCs and pMSC-exos groups had different densities of hair regenerated on DPW 28; the degree of hair regeneration in both pMSCs and pMSC-exos groups was comparable, but was higher than that of the aMSC-exos group (Figure 2B). These results suggest that pMSC-exos can improve the wound healing rate and inhibit scarring.

### 3.3 pMSC-exos promote the regeneration of cutaneous appendages

**FIGURE 3:**
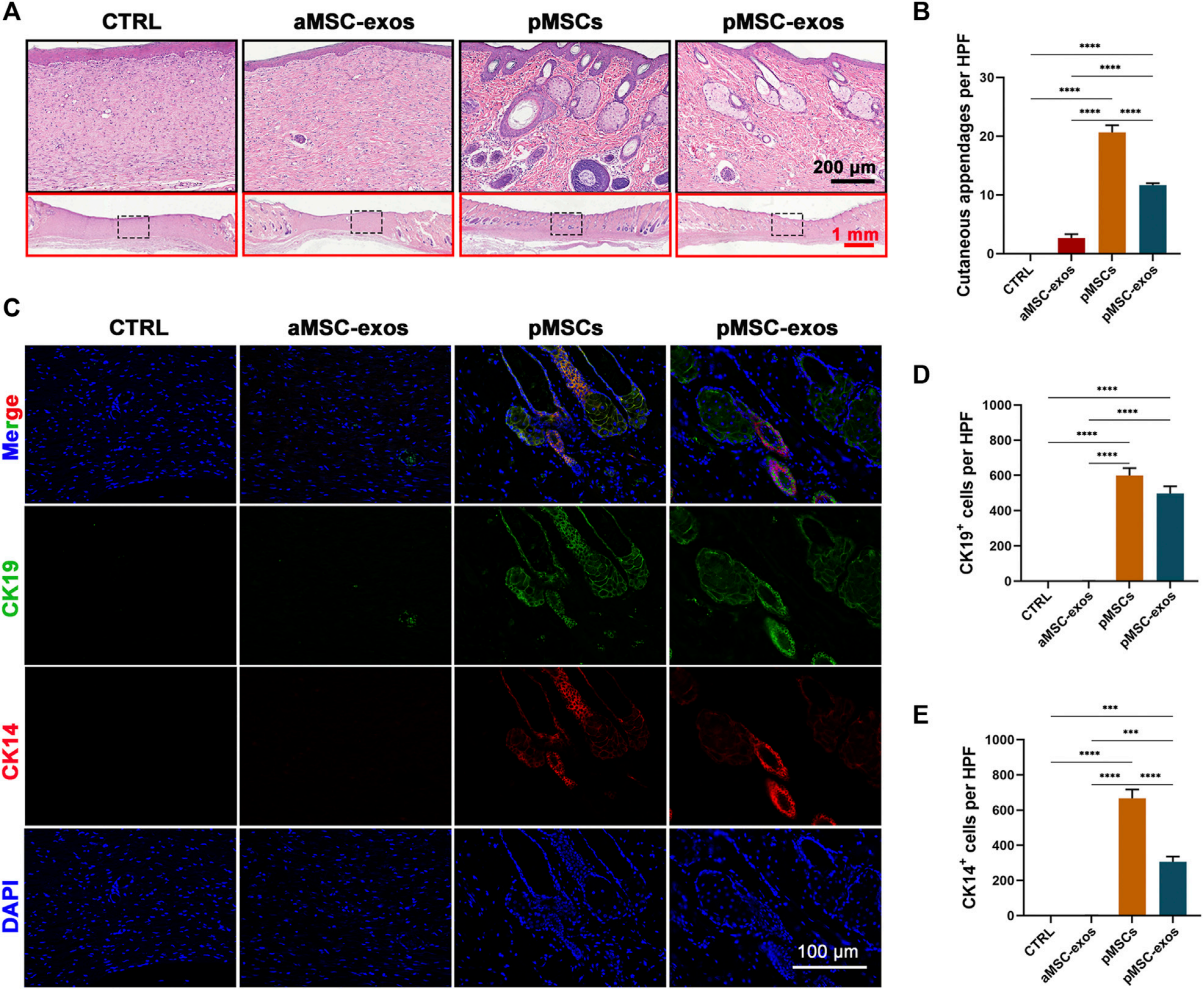
pMSC-exos promote the regeneration of cutaneous appendages. **(A)** HE staining of the healed skin. **(B)** Number of cutaneous appendages per HPF in the healed skin according to HE staining. **(C)** CK14 and CK19 IF staining. **(D,E)** Numbers of CK14^+^ cells and CK19^+^ cells per HPF according to IF staining. Mean ± SEM; ***p < .001, ****p < .0001; n = 3. HPF, 20× high-power field; CK14, cytokeratin 14; CK19, cytokeratin 19.

After evaluating the effect of pMSC-exos on wound healing rate and scar formation in appearance, the regeneration level of cutaneous appendages was further evaluated. Results of HE staining showed no regenerated cutaneous appendage was observed in the CTRL group, whereas, regenerated glands and hair follicles were frequently encountered in the healed skin of the pMSCs and pMSC-exos groups (Figure 3A). The number of cutaneous appendages per 20× HPF in the CTRL, AMSCs, pMSCs, and pMSC-exos were 0.00 ± 0.00, 2.67 ± 0.94, 20.67 ± 1.70 and 11.67 ± 0.47, respectively (Figure 3B). Next, immunofluorescence (IF) staining was performed to visualize the expression of specific markers CK19 (sebaceous glands) and CK14 (hair follicles). As expected, no CK19^+^ cells and CK14^+^ cells were observed in healed skin of the CTRL group; in contrast, numerous CK19^+^ cells and CK14^+^ cells were observed in the pMSC-exos group, although the number of CK19^+^ cells was significantly less than in the pMSCs group (*p* < .0001, Figures 3C–E). Besides, the numbers of CK19^+^ cells and CK14^+^ cells in the pMSC-exos group were significantly higher than in the aMSC-exos group (*p* < .0001, Figures 3C–E). The results suggest that the pMSC-exos treatment can effectively promote cutaneous appendage regeneration in wound healing in rats, and the effects can be comparable to pMSCs in some aspects and stronger than aMSC-exos.

### 3.4 pMSC-exos regulate collagen distribution in wound healing

**FIGURE 4:**
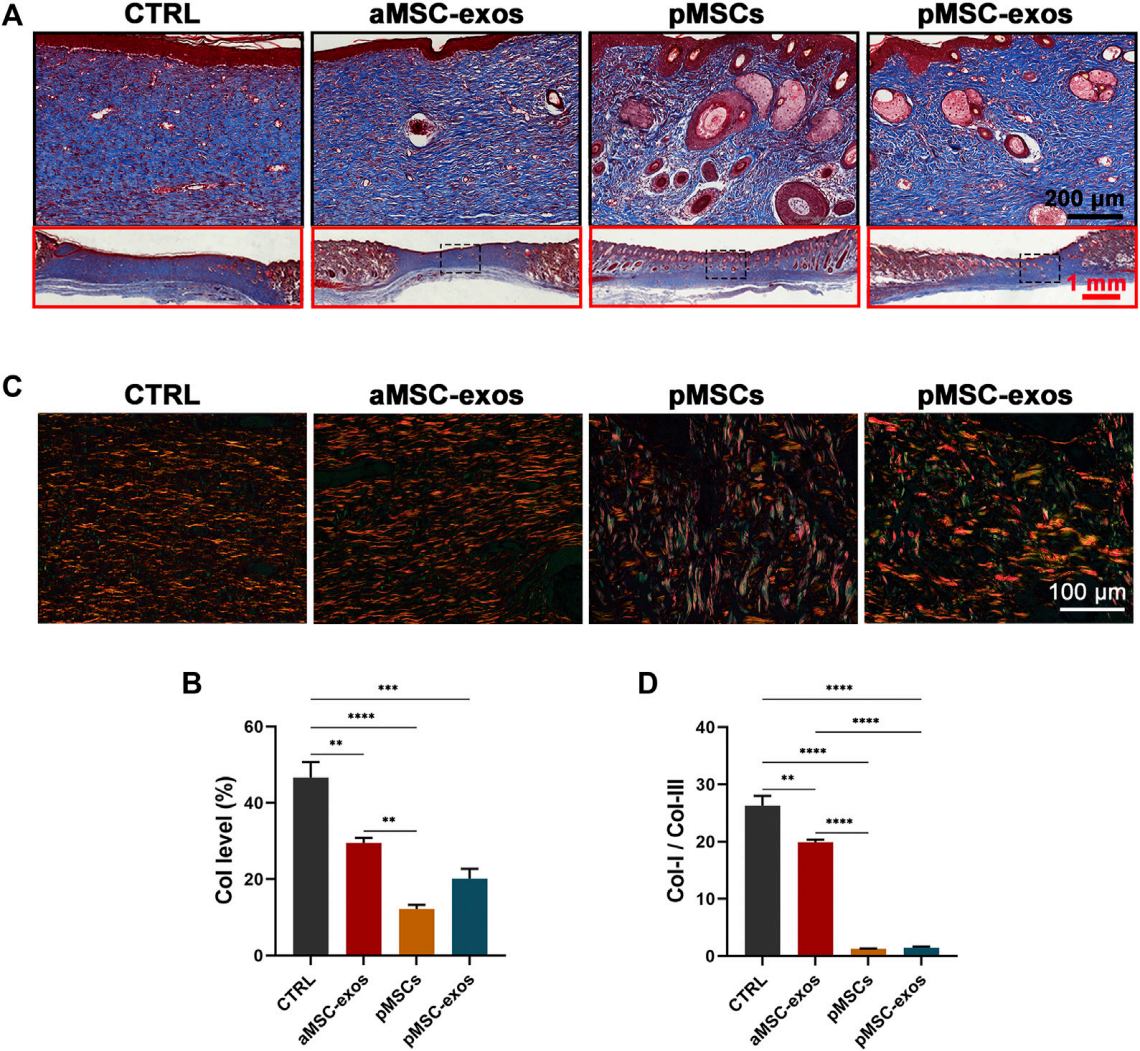
pMSC-exos regulate the structure and composition of collagen in wound healing. **(A)** Masson staining of the healed skin. **(B)** Accounts of collagen fibers (blue area) in the healed skin according to Masson staining. **(C)** Sirius red staining of the healed skin. **(D)** The ratio of collagen I (red area) to collagen III (green area) in the healed skin according to Sirius red staining. Mean ± SEM; **p < .01, ***p < .001, ****p < .0001; *n* = 3. Col, collagen.

The collagen level and structural composition are critical factors for scarring and regeneration in wound healing, so they were analyzed *via* Masson and Sirius red stainings. Results of Masson staining showed that collagen fibers (blue area) were abundantly accumulated in healed skin of the CTRL group, whereas pMSC-exos treatment significantly reduced the level of collagen fibers (Figures 4A, B; *p* < .001). However, no significant difference in the level of collagen fibers between the pMSC-exos and aMSC-exos or pMSCs group. Interestingly, the Sirius red staining showed that levels of collagen I (red area) in the CTRL and aMSC-exos groups were higher than that in the pMSCs and pMSC-exos groups (Figure 4C). In contrast, levels of collagen III (green area in Sirius red staining) in the CTRL and aMSC-exos groups were lower than that in the pMSCs and pMSC-exos groups (Figure 4C). The ratio of collagen I to collagen III of the pMSC-exos group was significantly lower than those of the CTRL and aMSC-exos groups (*p* < .0001). In contrast, there was no significant difference between the pMSCs and pMSC-exos groups (Figure 4D). Overall, these results suggest that the level of total collagen fibers was reduced, collagen I (pro-scar formation) was down-regulated and collagen III (anti-scar formation) was up-regulated by pMSC-exos.

### 3.5 pMSC-exos improve regeneration of blood vessels

**FIGURE 5:**
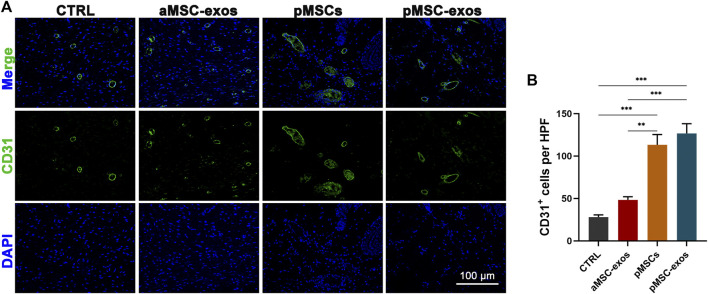
pMSC-exos improve regeneration of blood vessels in wound healing. **(A)** CD31 IF staining of the healed skin. **(B)** Number of CD31^+^ cells per HPF according to IF staining. Mean ± SEM; **p < .01, ***p < .001; *n* = 3.

IF staining was performed to visualize the expression of specific markers CD31 of vascular endothelial cells. The results showed that the number of CD31^+^ cells in healed skin in the pMSC-exos group was significantly higher than that in the CTRL and aMSC-exos groups (*p* < .001; Figures 5A, B). However, no significance of CD31^+^ cell number was observed in healed skin in the pMSC-exos and pMSCs groups, suggesting that the effects on the regeneration of blood vessels of pMSC-exos can be comparable to pMSCs.

### 3.6 pMSC-exos prevent EN1 activation

**FIGURE 6:**
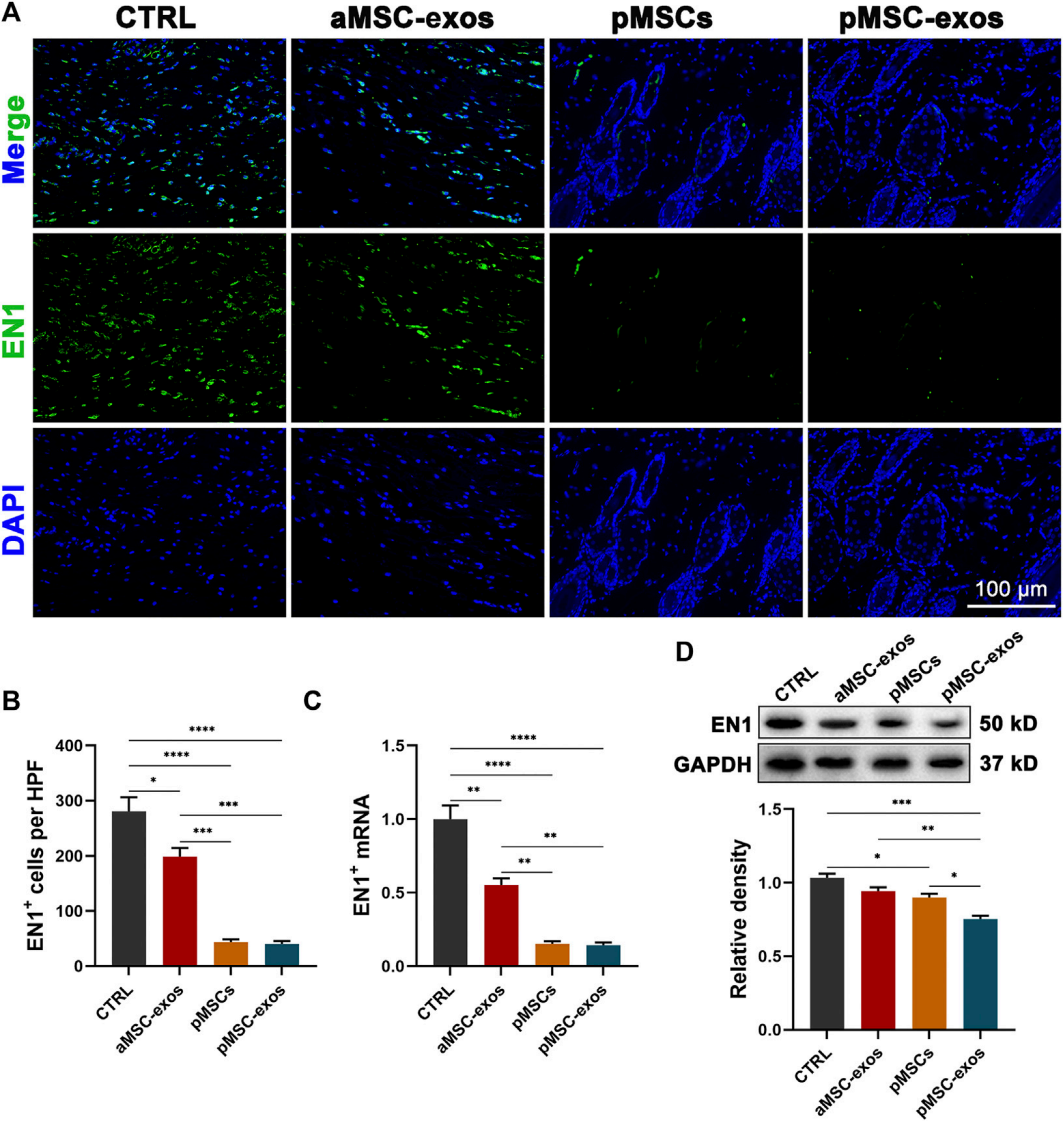
pMSC-exos prevent EN1 activation in wound healing. **(A)** EN1 IF staining of the healed skin. **(B)** Number of EN1^+^ cells per HPF according to IF staining. **(C)** mRNA levels in the healed skin via qRT-PCR. **(D)** Protein (EN1) levels in the healed skin via western blot. Mean ± SEM; *p < .05, **p < .01, ***p < .001, ****p < .0001; *n* = 3. EN1, Engrailed-1.

To further reveal the mechanism of wound regeneration induced by pMSC-exos, the level of EN1 (reported to play a critical role in scarring (Rinkevich et al., 2015; Mascharak et al., 2021)) in wound healing in healed skin was detected. Results of IF staining showed that a large number of EN1^+^ cells were observed in the CTRL group. Whereas pMSC and pMSC-exos treatments significantly reduced the number of EN1^+^ cells (*p* < .0001; Figures 6A, B), and the effects of pMSC and pMSC-exos were all stronger than the treatment of aMSC-exos (*p* < .001; Figures 6A, B). The change in mRNA level of EN1 was further verified using qRT-PCR, and showed similar trends to the IF results. Consistently, pMSCs and pMSC-exos treatments also significantly decreased the mRNA and protein levels of EN1 (Figures 6C, D). These results suggest that pMSC-exos can effectively inhibit EN1 expression; thus, pMSC-exos induced wound regeneration may be achieved mainly via inhibiting EN1 activation.

**FIGURE 7:**
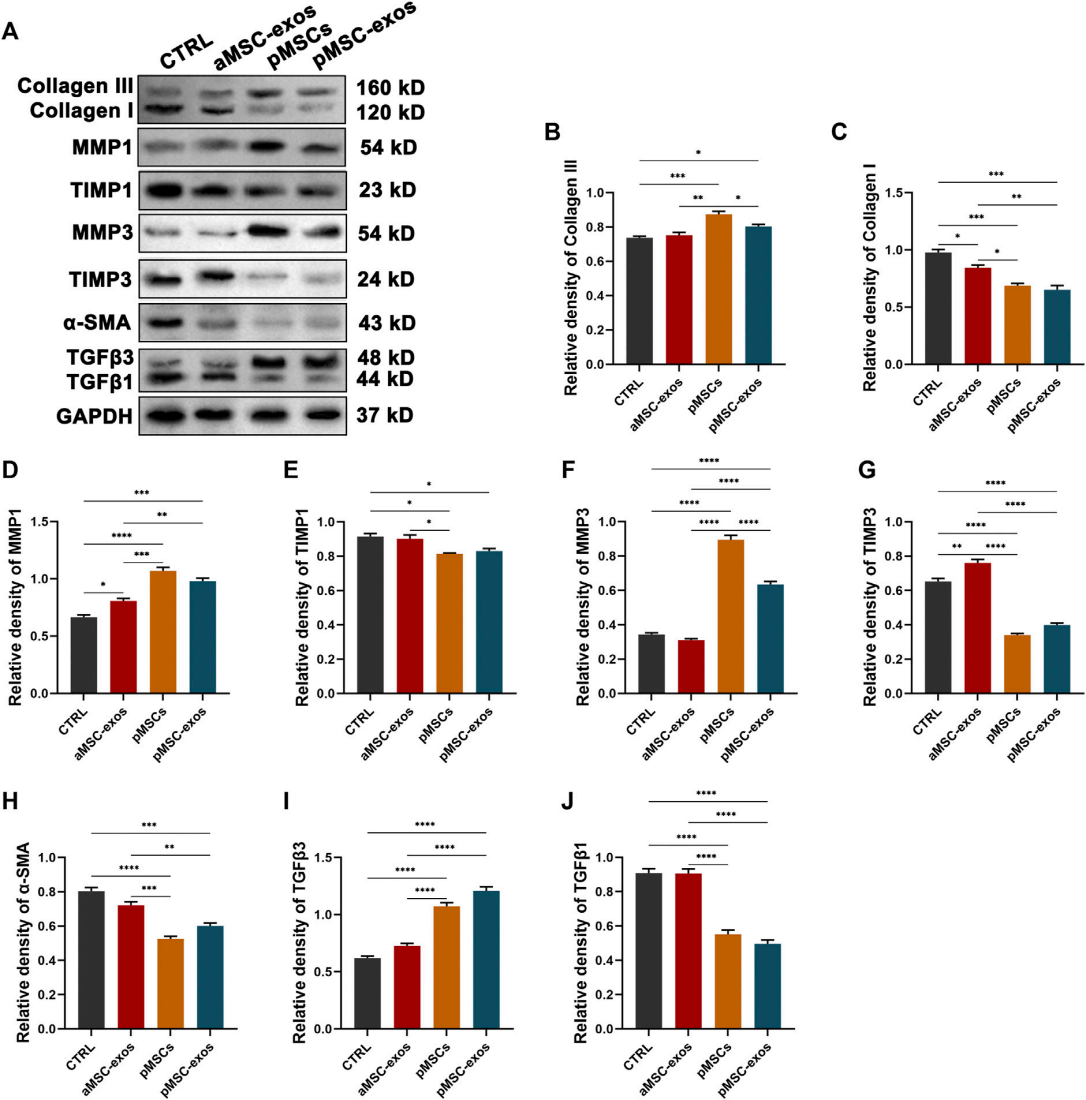
pMSC-exos improve the expressions of fibroblast and ECM remodeling associated genes in wound healing. **(A)** Protein (collagen III, collagen I, MMP1, TIMP1, MMP3, TIMP3, and α-SMA) levels in the healed skin via western blot. **(B–J)** Relative density of protein bands in **(A)**. Mean ± SEM; *p < .05, **p < .01, ***p < .001, ****p < .0001; *n* = 3. MMP, matrix metalloproteinase; TIMP, tissue inhibitor of metalloproteinase; α-SMA, α-smooth muscle actin, TGFβ, transforming growth factor-β.

Additionally, the impact of pMSC-exo treatment on fibroblast and ECM inducing/remodeling-associated genes, including α-SMA, collagen I, collagen III, MMP1, MMP3, TIMP1, TIMP3, TGFβ1, and TGFβ3, were also evaluated in the healed skin through western blot analysis. The results showed that genes anti-scar formation (collagen III, MMP1, MMP3, and TGFβ3) were significantly up-regulated, whereas those pro-scar formation (collagen I, TIMP1, TIMP3, α-SMA, and TGFβ1) down-regulated treated by pMSC-exos (Figure 7).

## 4 Discussion

Adult wound healing generally results in scarring, mainly caused by the activation of EN1 of fibroblasts (Rinkevich et al., 2015; Mascharak et al., 2021). MSC treatment has recently been recognized as a promising strategy for inducing wound regeneration (Ojeh et al., 2015; Fang et al., 2016; Duan et al., 2020). pMSCs have been used to repair injuries to the spinal cord (Jiang et al., 2017), heart (Huynh, 2019), skeleton (Longo et al., 2012), *etc.* However, few attempts have been made to study the effects of pMSCs on inducing wound regeneration. Studies have shown that MSCs perform their functions mainly through their paracrine, but there are few studies on the function of pMSC-exos. For the first time, the present study revealed that pMSCs accelerate wound healing rate and improve healing quality in wound healing, which can be, in part, *via* exosomes. We further demonstrated that pMSC-exos induced wound regeneration may achieve *via* inhibiting EN1^+^ fibroblast activation. To a certain extent, pMSC-exos could be served as a new strategy to stimulate wound regeneration in the clinic.

In recent decades, MSC-based therapies have shown proven advantages in wound closure and cutaneous appendage regeneration (Zhang et al., 2021a; Zhang et al., 2021b). Studies have revealed that the potential of cell proliferation and differentiation is positively correlated with its therapeutic capacity. Therefore, the source of MSCs needs to be further expanded to maximize wound regeneration. The placenta is an acceptable and efficient source of MSCs. Compared with general adult MSC, pMSCs exhibit more advantages due to their stronger potential for cell proliferation and differentiation (Yi et al., 2020; Maraldi and Russo, 2022). The function of MSCs in cell therapy on tissue injury may achieve through the paracrine process. As reviewed elsewhere, most of these effects are mediated by their exosomes, which are derived from adipose MSC (Hu et al., 2016), epidermal MSCs (Duan et al., 2020), umbilical cord MSC (Fang et al., 2016), and amniotic fluid MSCs (Zhang et al., 2021b) *via* sending the functional DNAs, miRNAs, and protein factors.

Exosomes present in MSC-conditioned medium are one of the major secretory products of MSCs, along with membrane-bound vesicles between 30 and 150 nm in diameter (Pegtel and Gould, 2019; Kalluri and LeBleu, 2020). Compared with MSCs, exosomes are easier to be stored and transported, and less risky for tumorigenicity and immune rejection (Wechsler et al., 2021). In this study, we prepared the exosomes from the pMSCs; and evaluated their effects on wound regeneration *via* a full-thickness wounded rat model. Expectedly, it found that pMSC-exos significantly reduced scarring and promoted appendage regeneration in this model. The effects were found to be more potent than aMSC-exos, and comparable to the pMSC treatment.

Collagen synthesis and its structural composition are critical for scarring and regenerative wound healing. Fibroblasts in regeneration (e.g., Fetal wound regeneration) differ from scarring in collagen synthesis in terms of speed of deposition and collagen types. Most known is the persistence of more collagen III and less collagen I in regeneration than in scarring. Duan et al. (2020) (Zhang et al., 2021b) reported that higher levels of collagen III yield smaller, reticular structures with more basket-weave than collagen I and contribute toward regenerative wound healing. In our study, the ratio of collagen I to collagen III of the pMSC-exos group was significantly lower than those of the CTRL and aMSC-exos groups (*p* < .0001). It is well known that TGFβ1 is a recognized fibrotic cytokine to induce scarring in wound healing; TGFβ3 is a pro-regeneration factor that is anti-scarring (e.g., Fetal wound regeneration) (Buchanan et al., 2009; Helmo et al., 2013). We found that TGFβ3 was significantly up-regulated, whereas TGFβ1 was down-regulated treated by pMSC-exos. ECM remodeling is another essential component in wound healing. It requires the coordinated regulation of MMPs and their inhibitors, the TIMPs (Satish and Kathju, 2010). Scarred wound healing is correlated with low MMP activity and high TIMP activity. Our study showed that MMP1 and MMP3 were significantly up-regulated, whereas TIMP1 and TIMP3 were down-regulated treated by pMSC-exos. Therefore, remodeling of the ECM structure in the healing tissue must have been promoted in the pMSC-exos group thus constituting a microenvironment that is permissive to cell migration and proliferation in wound healing. All the above findings suggest that wound healing treated by pMSC-exos represents a regeneration phenotype. The pMSC-exos may potentially be developed as a novel cell-free therapeutic for wound regeneration.

Fibroblasts are heterogeneous and include multiple subpopulations with distinct roles (Guerrero-Juarez et al., 2019). Wounding activates a dermal fibroblast subset to exhibit ECM production and contractile properties that result in scarring (Leavitt et al., 2020). Recently, Michael T. Longaker et al. identified a subpopulation of dermal fibroblasts defined by EN1 expression, activated after being wounded, and responsible for scarring (Rinkevich et al., 2015; Mascharak et al., 2021). Consequently, if any means can interrupt the EN1^+^ fibroblast activation, wound healing may be deflected from scarring to regeneration. Intriguingly, in the present study, pMSC-exos treatment on full thickness rat wounds significantly reduced the number of EN1^+^ fibroblasts; thus, the effects of pMSC-exos on wound regeneration are likely achieved through inhibiting EN1^+^ fibroblast activation.

The underlying molecular mechanism of pMSC-exos inhibiting EN1^+^ fibroblast activation can only be speculative, and the YAP signaling pathway is reported to play a critical role in EN1^+^ fibroblast activation (Ji et al., 2020; Mascharak et al., 2021). Besides, it was reported that activation of the YAP signaling pathway promotes renal fibrosis, pulmonary fibrosis and liver fibrosis and that YAP degradation treated by MSC-derived exosomes blocks the progression of fibrosis (Ji et al., 2020; Wang et al., 2021; Liang et al., 2022). Thus, it is possible that our findings regarding YAP signaling in skin scarring are relevant to fibrotic responses in other organs and could have similar therapeutic implications. pMSC-exos effectively inhibited expression of EN1 may be due to contain substances that can specifically target the YAP signaling pathway. It is known that the “cargos” carried by exosomes are crucial to their function, and miRNA is reported to be one of the most important substances (Pegtel and Gould, 2019). Xu et al. (2021) reported that pMSC-exos contained let-7, miR-210, miR-377, miR-195, miR-145 and miR-34a in high abundance. Interestingly, both let-7 and miR-195 specifically target YAP as predicted by Targetscan (https://www.targetscan.org/vert_72/). Moreover, previous studies have demonstrated that let-7, miR-210, miR-195 and miR-145 target the YAP signaling pathway. It is reported that let-7 could enhance cell apoptosis in trophoblasts through down-regulating YAP (Zha et al., 2020); miR-210-3p inhibits cholangiocarcinoma *via* down-regulating YAP (Jung et al., 2017); miR-195 suppresses the metastasis and epithelial-mesenchymal transition of hepatocellular carcinoma and by inhibiting YAP (Sun et al., 2017; Yu et al., 2017); miR-145 inhibits gastric cancer growth by reducing YES1-dependent YAP nuclear translocation (Sun et al., 2022). Besides miRNAs, protein components in the pMSC-exos are also considered to play a key role in inhibiting the YAP signaling pathway, which needs to be investigated in the future. Moreover, it has been reported that TGFβ1 also activate YAP in fibroblasts (Nakamura et al., 2021); thus, the inhibitory effect of pMSC-exos on the YAP signaling pathway may also be related to their targeted regulation of TGFβ1.

## 5 Conclusion

**FIGURE 8:**
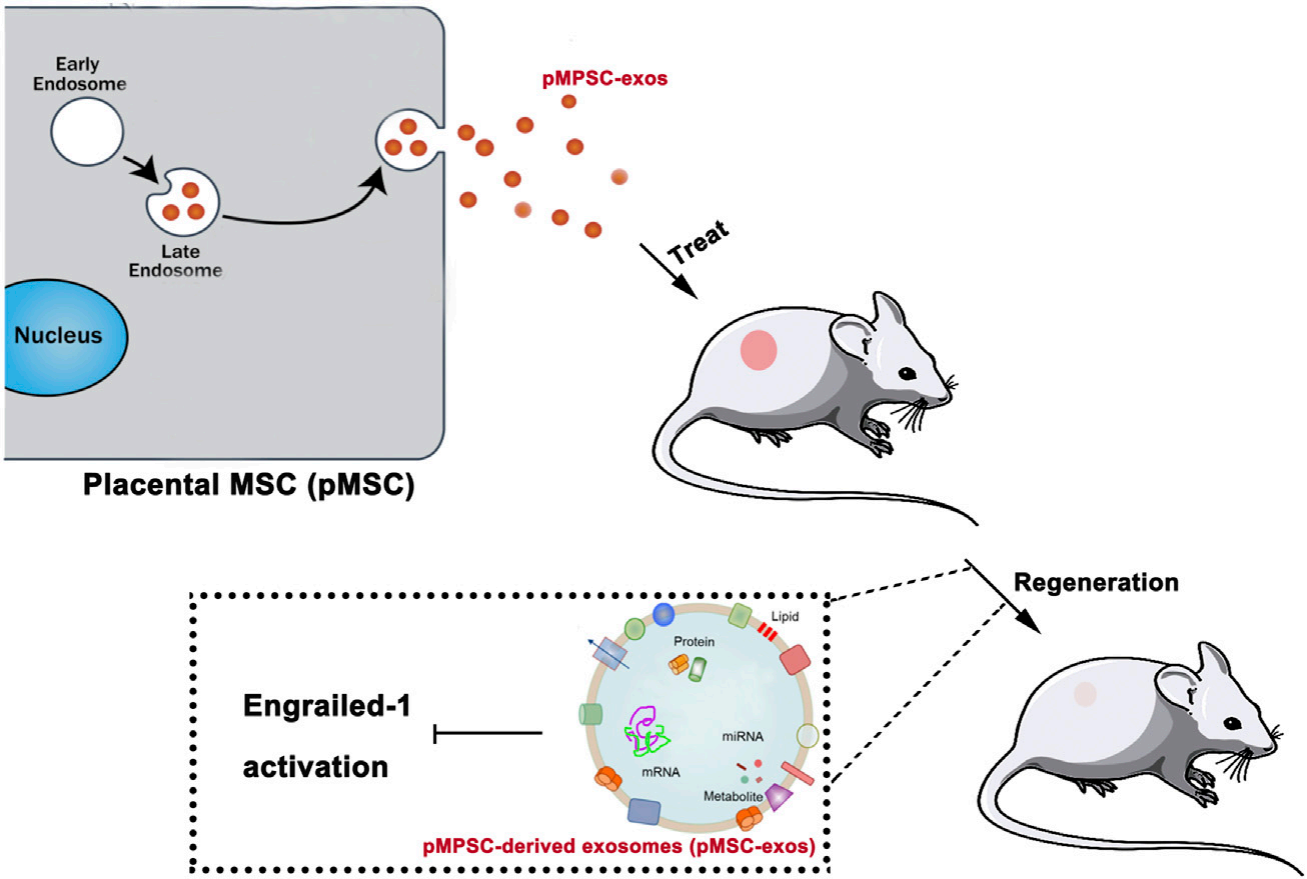
pMSC-exos induced wound regeneration via inhibiting Engrailed-1 activation. “Cargos” of pMSC-exos including DNAs, miRNAs, protein factors, *etc.* inhibit Engrailed-1 expression.

The present study revealed that pMSC-exos could effectively stimulate wound regeneration, and the effects can be comparable to pMSCs in some aspects and stronger than aMSC-exos. The effects of pMSC-exos in inducing wound regeneration may be mainly achieved *via* the down-regulation of the YAP signaling pathway, thus inhibiting the activation of Engraviled-1 (Figure 8). As an alternative to cell therapy, pMSC-exos might represent a novel approach to inducing wound regeneration in the clinic.

## Data Availability

The original contributions presented in the study are included in the article/Supplementary Material, further inquiries can be directed to the corresponding authors.
