# Supplementary material for: Placental stem cells-derived exosomes stimulate cutaneous wound regeneration via engrailed-1 inhibition
Source: Front Bioeng Biotechnol. 2022 Dec 9;10:1044773. doi: 10.3389/fbioe.2022.1044773 (PMC9780460; doi:10.3389/fbioe.2022.1044773)
Supplement: Supplementary file 1 [file Table2.DOCX]

Supplementary Material

**Table S1** Antibodies

| **Antibody** | **Company** | **Catalog number** | **Dilution** |
| --- | --- | --- | --- |
| CD9 | Beyotime, China | AF1192 | 1:2000 (WB) |
| CD34 | Beyotime, China | AF1387 | 1:500 (IF) |
| CD45 | Beyotime, China | AF7839 | 1:500 (IF) |
| CD63 | Beyotime, China | AF1471 | 1:2000 (WB) |
| CD73 | Bioss, China | bs4834R | 1:500 (IF) |
| CD90 | Bioss, China | bs0778R | 1:500 (IF) |
| CD105 | Bioss, China | bs0579R | 1:500 (IF) |
| CK14 | ProteinTech, China | 10143-1-AP | 1:500 (IF) |
| CK19 | ProteinTech, China | 10712-1-AP | 1:500 (IF) |
| Collagen I | ProteinTech, China | 14695-1-AP | 1:3000 (WB) |
| Collagen III | ProteinTech, China | bs0549R | 1:2000 (WB) |
| Engrailed-1 | Bioss, China | bs-11744R | 1:500 (IF)  1:2000 (WB) |
| GAPDH | Beyotime, China | AF2189 | 1:2000 (WB) |
| MMP1 | ProteinTech, China | 10371-2-AP | 1:3000 (WB) |
| MMP3 | ProteinTech, China | 17873-1-AP | 1:2000 (WB) |
| TIMP1 | ProteinTech, China | 16644-1-AP | 1:3000 (WB) |
| TIMP3 | ProteinTech, China | 10858-1-AP | 1:1000 (WB) |
| anti-TGFβ3 | Beyotime, China | AF8142 | 1:2000 (WB) |
| anti-TGFβ1 | Bioss, China | bsm-33345M | 1:3000 (WB) |
| Goat anti-Ms IgG/Cy3 | ProteinTech, China | SA00009-1 | 1:100 (IF) |
| Goat anti-Rb IgG/Cy3 | ProteinTech, China | SA00009-2 | 1:100 (IF) |
| Goat anti-Ms IgG/AF488 | ProteinTech, China | SA00013-1 | 1:100 (IF) |
| Goat anti-Rb IgG/AF488 | ProteinTech, China | SA00013-2 | 1:100 (IF) |
