# Supplementary material for: Placental stem cells-derived exosomes stimulate cutaneous wound regeneration via engrailed-1 inhibition
Source: Front Bioeng Biotechnol. 2022 Dec 9;10:1044773. doi: 10.3389/fbioe.2022.1044773 (PMC9780460; doi:10.3389/fbioe.2022.1044773)
Supplement: Supplementary file 2 [file DataSheet1.PDF]

Figure 1A

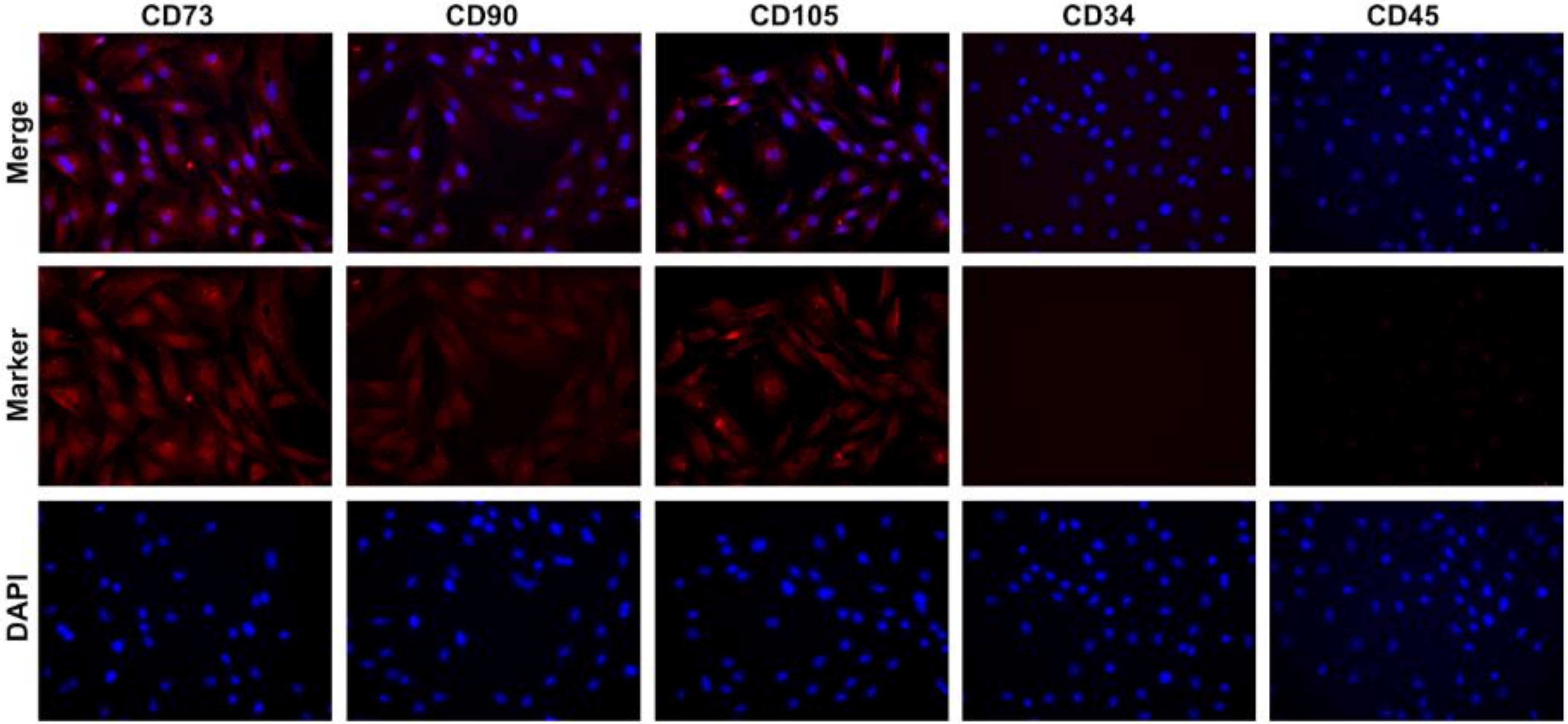

Figure 1B

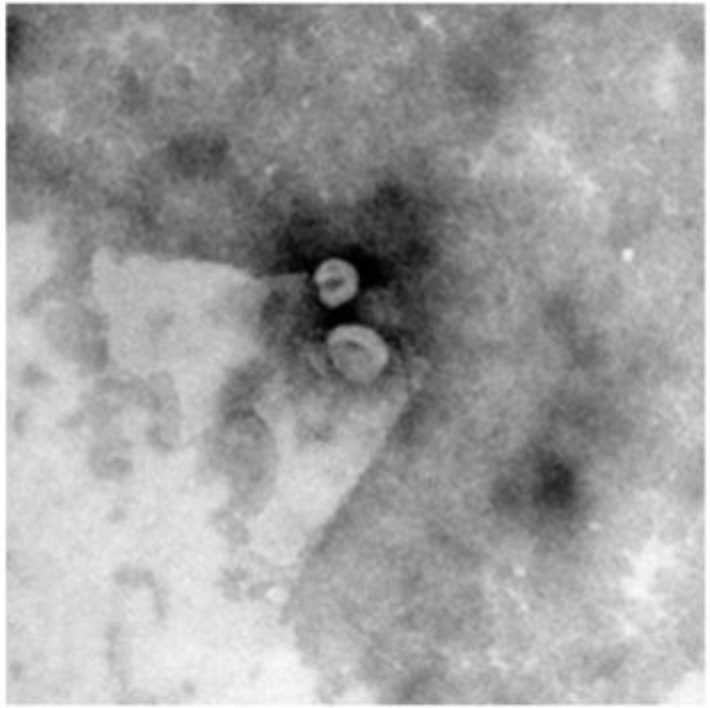

Figure 1D

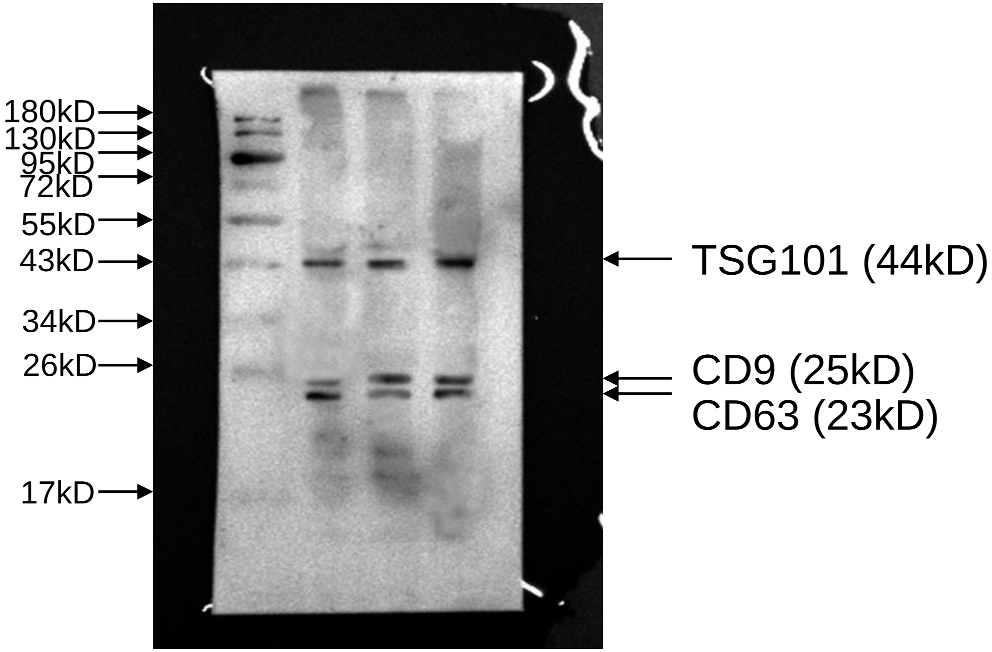

Figure 2B

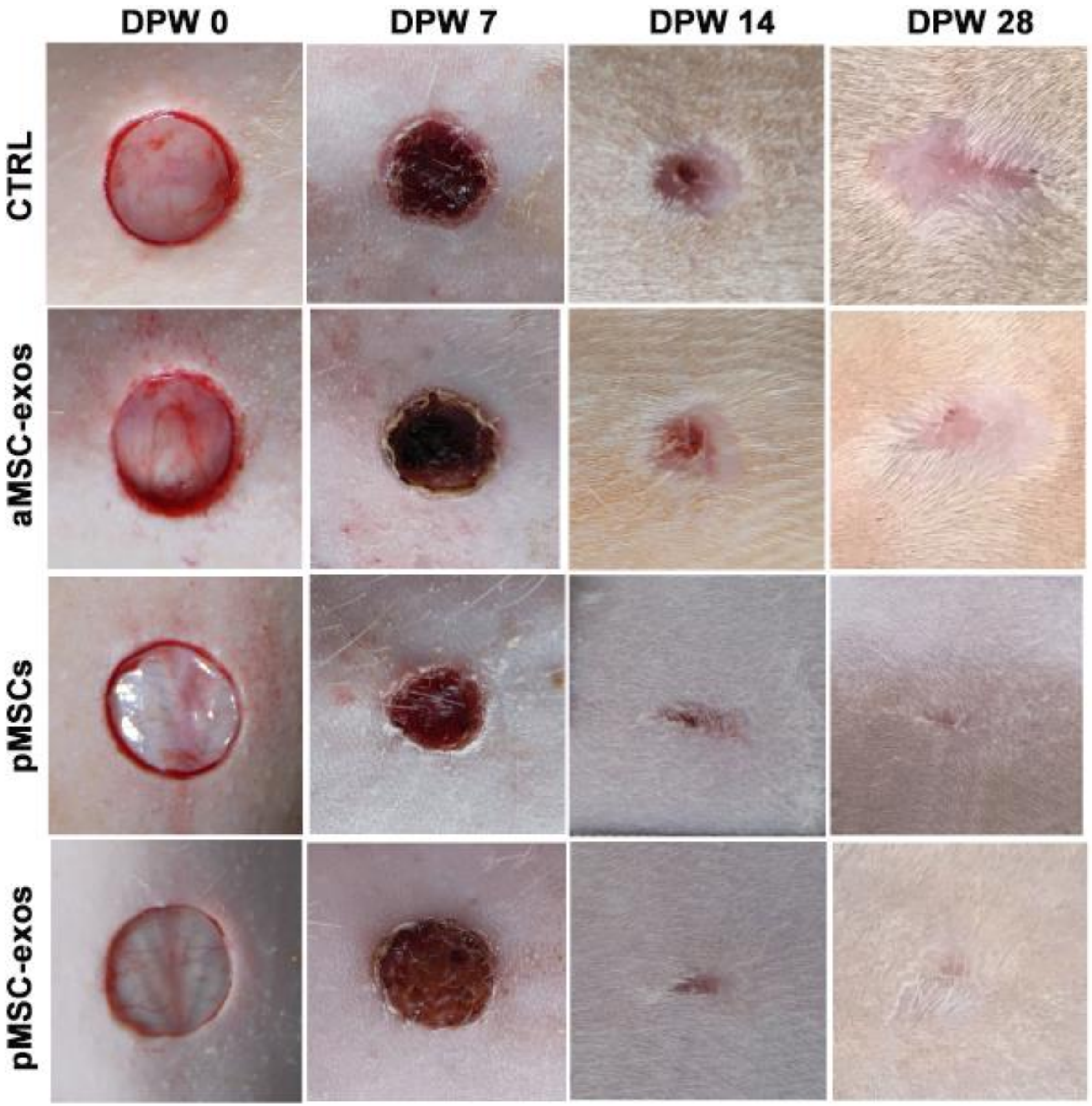

Figure 3A

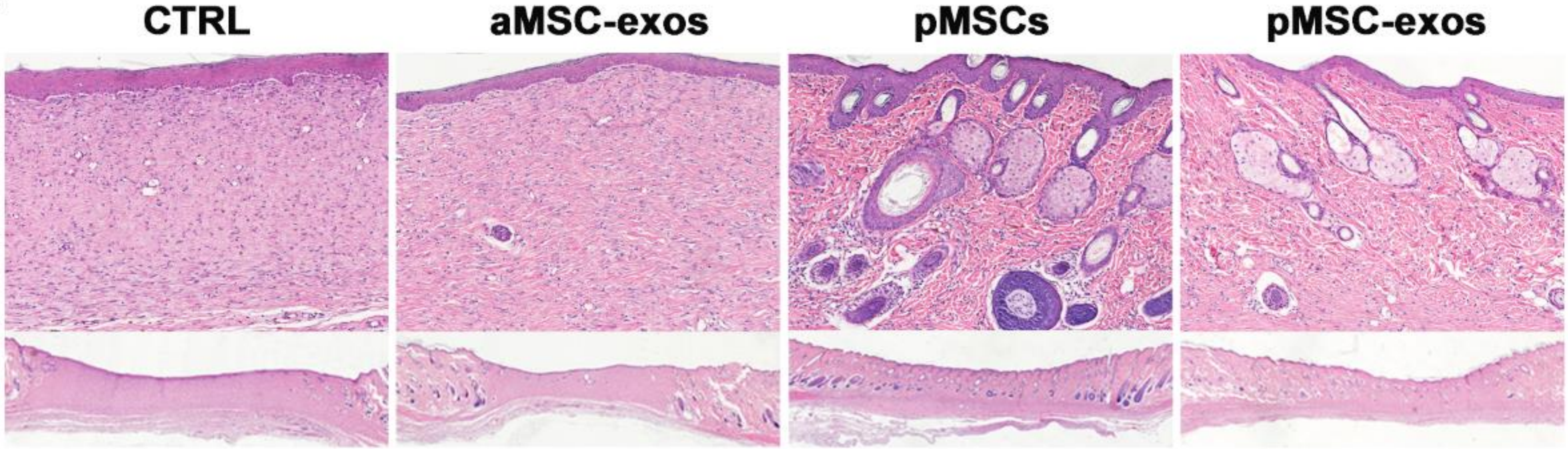

Figure 3C

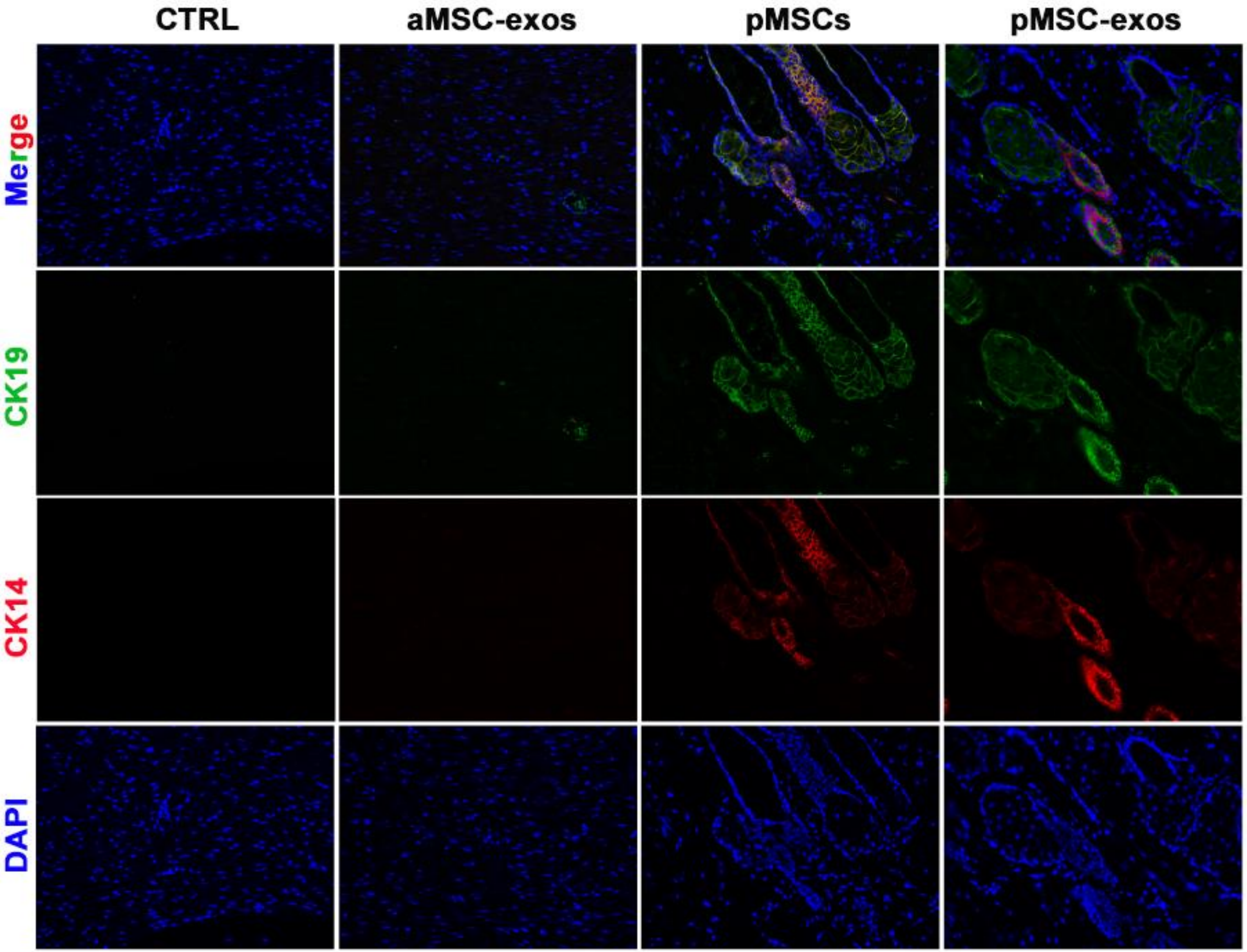

Figure 4A

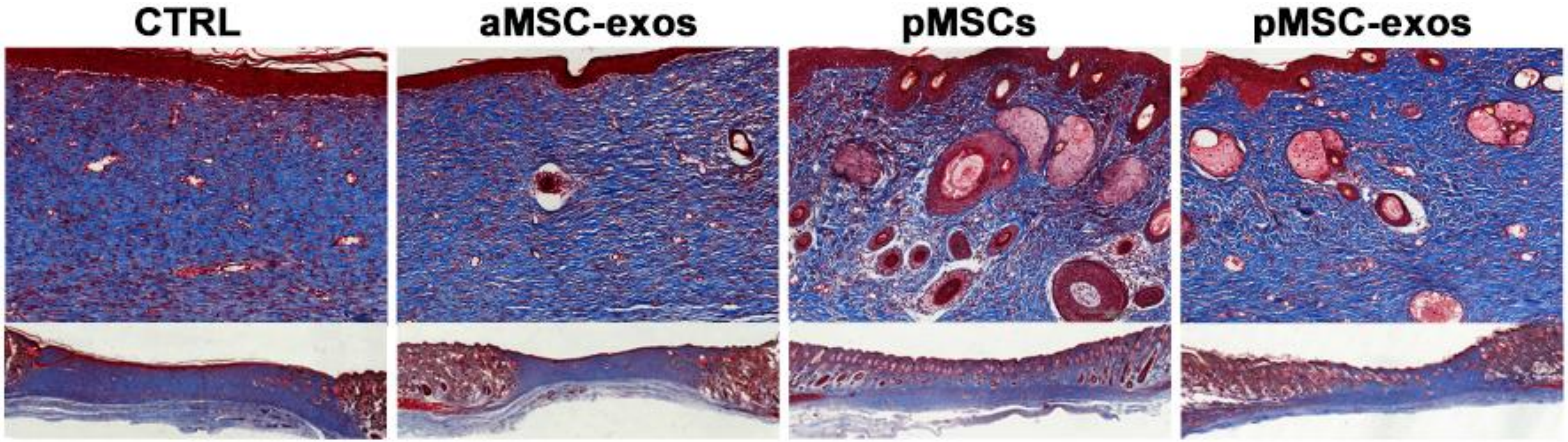

Figure 4C

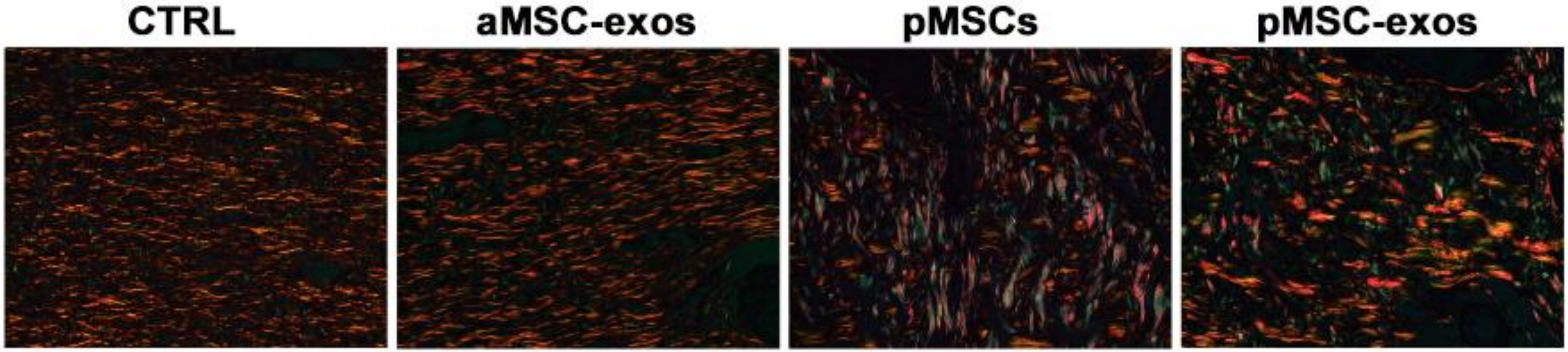

Figure 5A

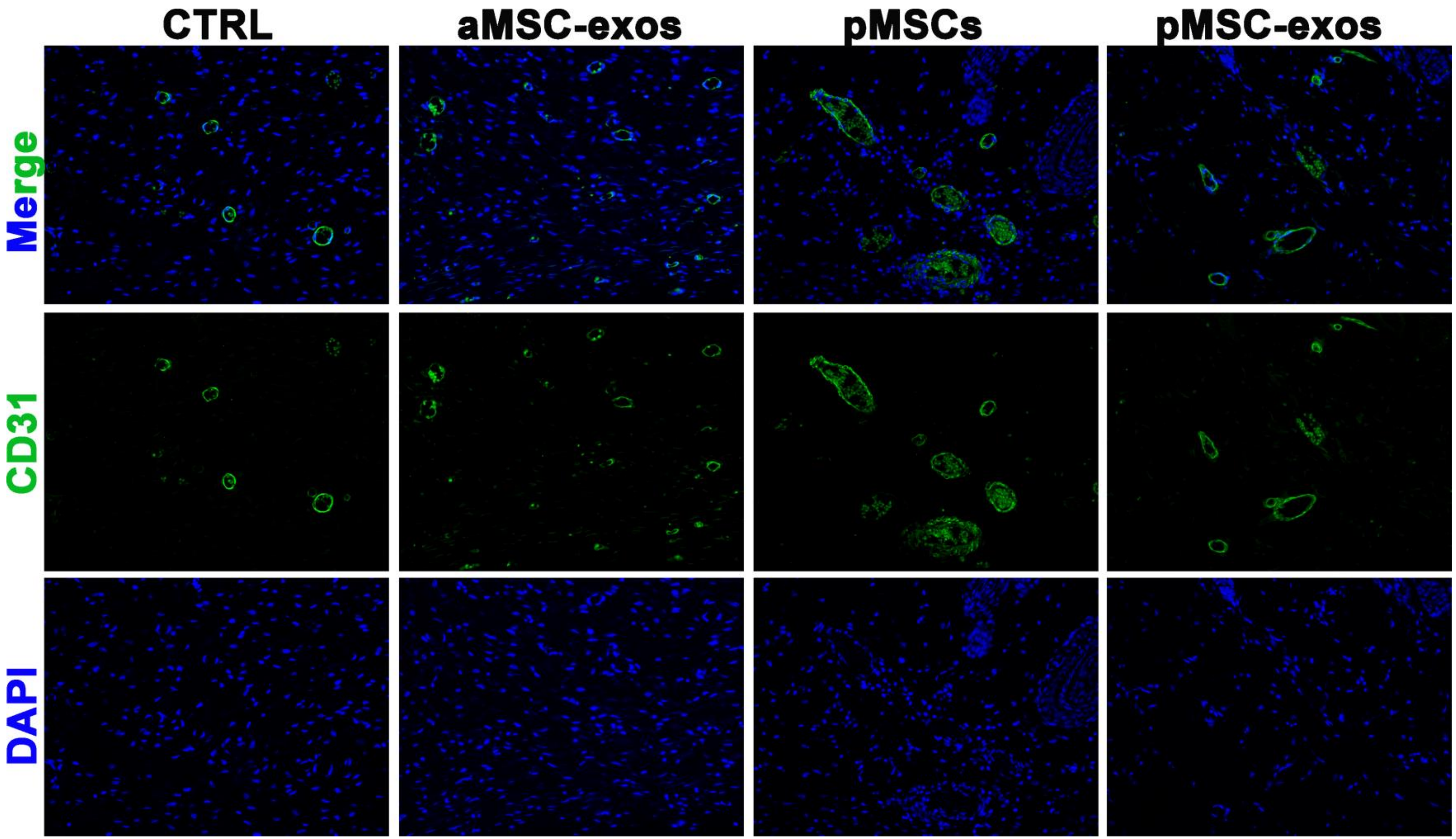

Figure 6A

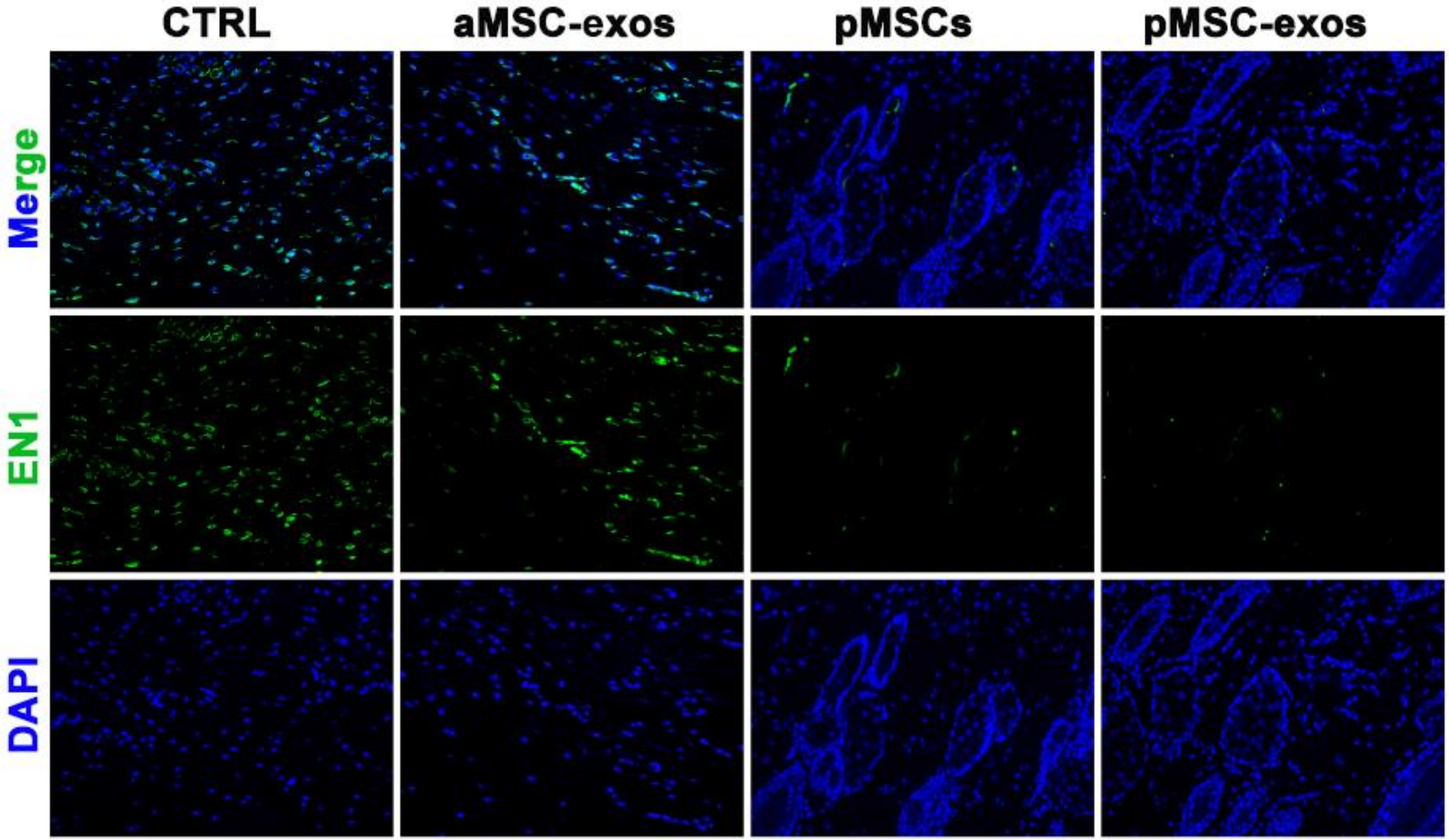

Figure 6D

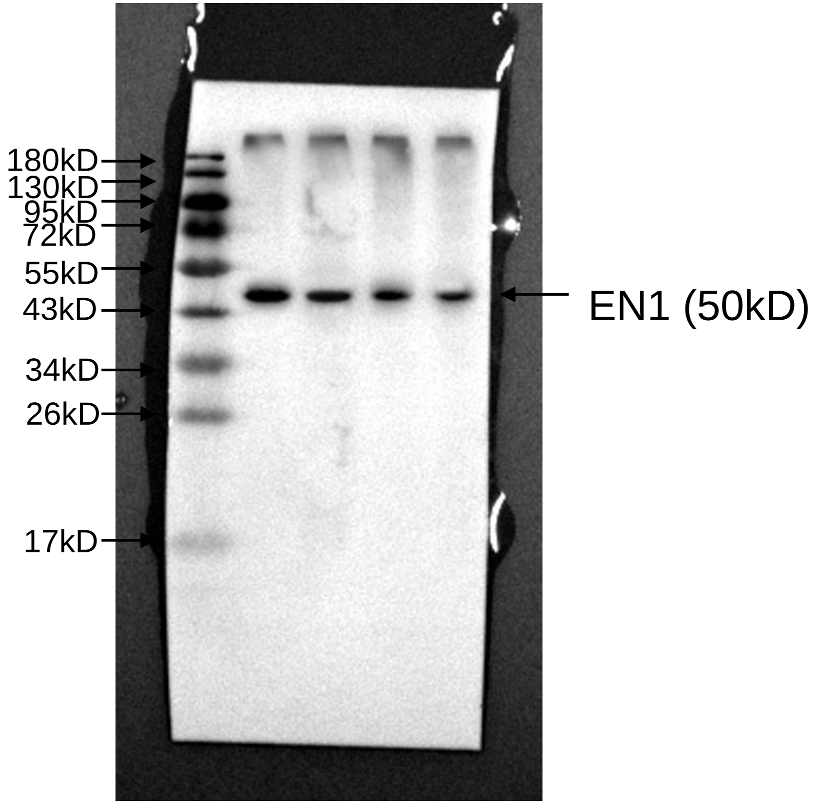

180kD
130kD
95kD
72kD
55kD
43kD
34kD
26kD
17kD
EN1 (50kD)

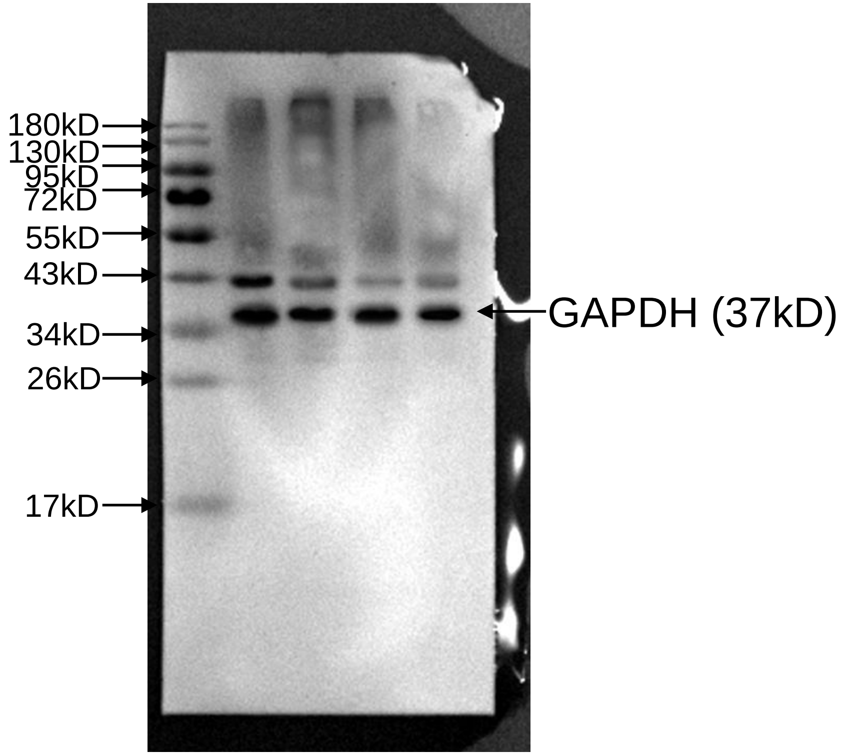

180kD
130kD
95kD
72kD
55kD
43kD
34kD
26kD
17kD
GAPDH (37kD)

Figure 7A

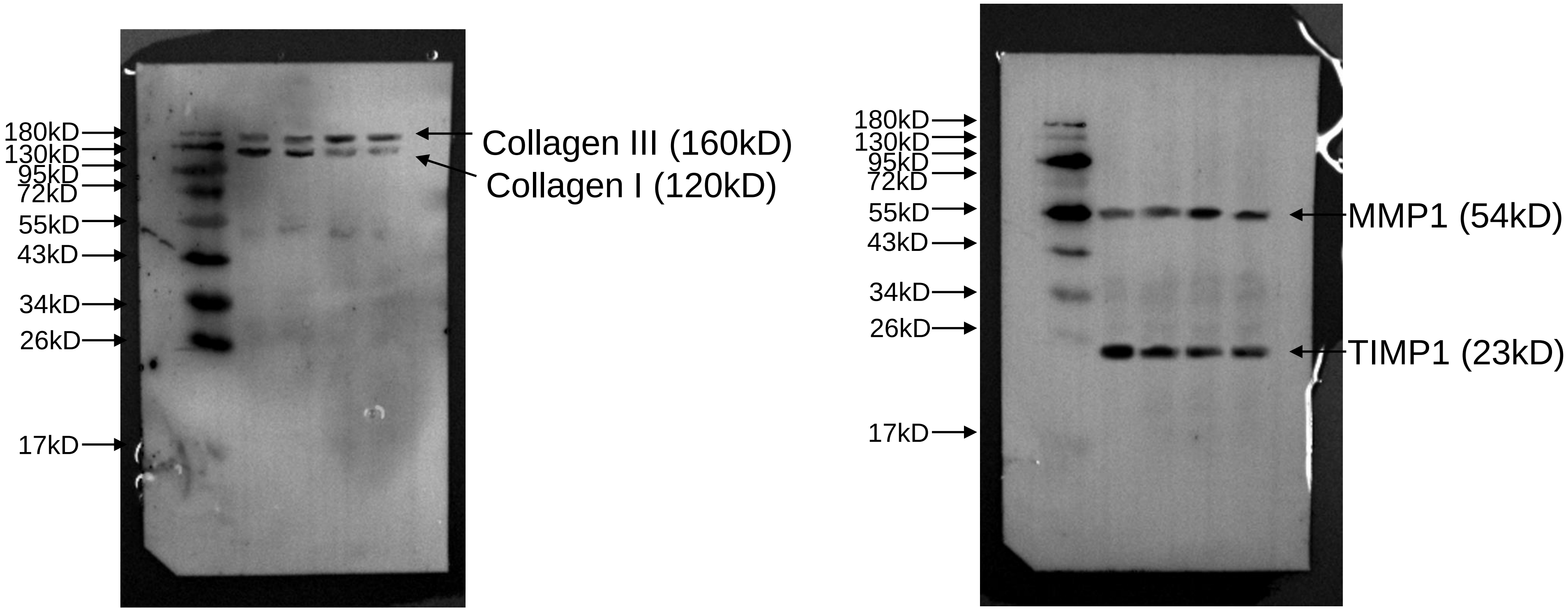

180kD
130kD
95kD
72kD
55kD
43kD
34kD
26kD
17kD
Collagen III (160kD)
Collagen I (120kD)
180kD
130kD
95kD
72kD
55kD
43kD
34kD
26kD
17kD
MMP1 (54kD)
TIMP1 (23kD)

Figure 7A

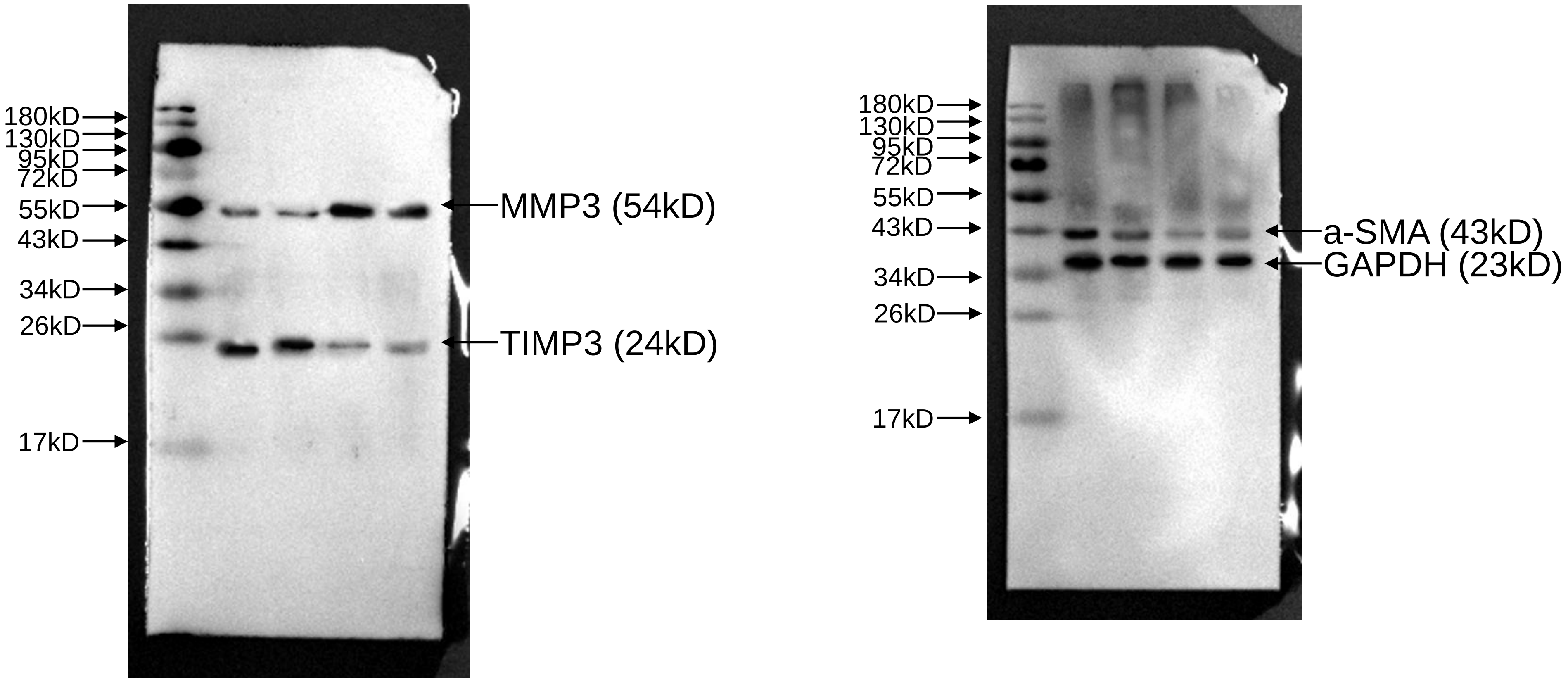

180kD
130kD
95kD
72kD
55kD
43kD
34kD
26kD
17kD
MMP3 (54kD)
TIMP3 (24kD)
180kD
130kD
95kD
72kD
55kD
43kD
34kD
26kD
17kD
a-SMA (43kD)
GAPDH (23kD)

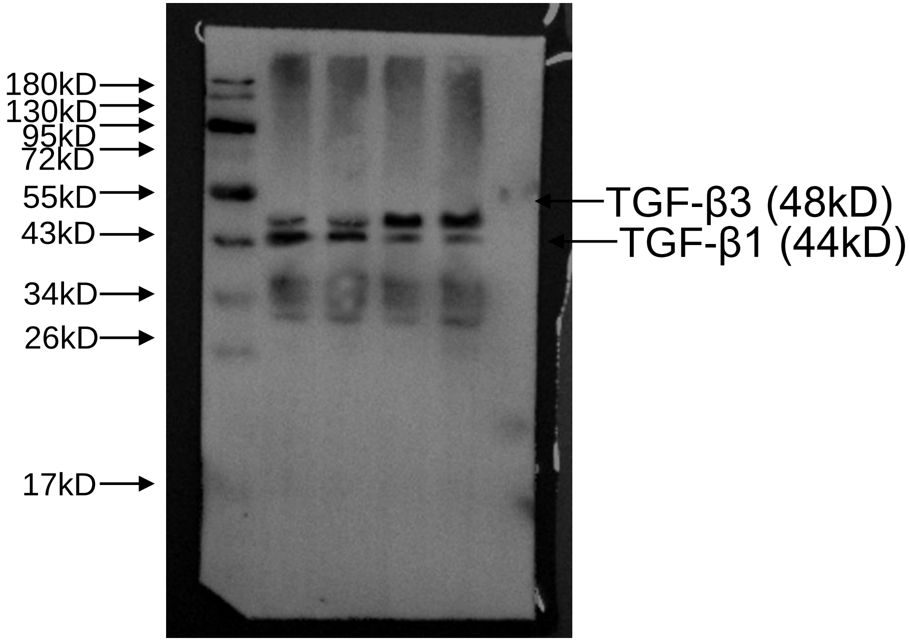

180kD
130kD
95kD
72kD
55kD
43kD
34kD
26kD
17kD
TGF-β3 (48kD)
TGF-β1 (44kD)
